# New mouse model based on adenocarcinoma 4T1 cells expressing HPV16 E6 and E7 applied to assess the efficacy of therapeutic and prophylactic E6/E7-based HPV16 vaccines

**DOI:** 10.21203/rs.3.rs-6328033/v1

**Published:** 2025-05-21

**Authors:** Juris Jansons, Daria Avdoshina, Alesja Dudorova, Elena Royo Rubio, Liba Sokolovska, Dmitry Perminov, Ilze Lindenberga, Hannes Nicolai, Svetlana Gebrila, Sona Chowdhury, Dace Skrastina, Jurijs Nazarovs, Joel M Palefsky, Maria Isaguliants

## Abstract

Background Development of immunotherapies and vaccines to treat HPV16-associated cancer requires reliable/effective small animal models. We developed such a model based on the murine mammary gland adenocarcinoma cells engineered to express HPV16 oncoproteins E6 and E7, and used it to assess the protective and therapeutic potential of E6/E7-based DNA-immunogens. Methods 4T1luc2 subclones with single genomic inserts of HPV16 E6/E7 DNA (B2, H6) were obtained by lentiviral transduction. DNA-immunogens were designed encoding expression-optimized consensus HPV16 E6 and E7 mutated to disrupt p53- and Rb-binding, both controlled by the human elongation factor 1a promoter. In prophylactic settings, BALB/c mice received E6, E7, E6/E7 DNA or vector, followed by challenge with B2 or H6 cells, and in therapeutic settings, were challenged with B2 or H6 cells, and DNA-immunized with E6 or vector. In reference series, C57bl/6 mice were challenged with TC1/luc2 cells and DNA-immunized with E6, E7, or E6/E7, or vector DNA. Tumor growth was monitored morphometrically and by *in vivo* bioluminescence imaging (BLI); metastatic activity, by *ex vivo* organ BLI, PCR and histology, and *in vitro* cytokine production by T-cells of immunized mice, by flow cytometry. Results E6/E7-expressing 4T1luc2 subclones B2 and H6 longitudinally expressed mRNA of E7 and of E6*I, E6*II, full length E6 (E6FL) isoforms. The levels of expression of E6 and E7 mRNA significantly increased with time. In naïve mice, B2 and H6 generated solid tumors with lung metastases. B2 and H6 cells were used to assess the efficacy of prophylactic DNA-immunization with E6 and E7. In immunogenicity tests, E6 DNA recipients developed Th1-type T-cell response, their unstimulated T-cells produced IFN-g and IL-2. E7 DNA was nonimmunogenic, while unstimulated T-cells produced TNF-α. In prophylactic settings, DNA-immunization with E6 and E7 suppressed formation of B2/H6 tumors. In therapeutic settings, DNA-immunization with E6 (not E7) restricted growth of TC-1/luc2 tumors, but had no effect on tumorigenic or metastatic activity of E6/E7-expressing 4T1luc2 cells. In both TC-1/luc2 and 4T1luc2E6/E7-models, E7 DNA recipients developed systemic inflammation with liver injury. Conclusions 4T1luc2 cells stably expressing HPV16 E6/E7 present an attractive alternative to TC-1 model allowing stringent assessment of both protective and therapeutic potential of E6/E7-based vaccines in BALB/c mice.

### 1. INTRODUCTION

Infection of mucosa with high risk HPV types (HR HPVs) causes 5% of all cancers worldwide including cervical cancer, the majority of other anogenital cancers, and large proportion of head and neck cancers [1]. Although HPV prophylactic vaccination prevents almost 90% of the HPV-associated infections, global vaccination coverage is still insufficient leading to millions of cases of chronic HPV infection [2, 3] with subsequent malignant transformation of epithelial cells, and development of neoplasia and cancer [4]. One of the instruments to treat HPV-associated malignancies could be the therapeutic HPV vaccination alone and in combination with chemotherapy and/or immunotherapy [5]. Multiple clinical trials of HPV16 therapeutic vaccines have generated promising, although not definite, results including regression of the neoplastic lesions [6]. Furthermore, many vaccine candidates that showed promise in preclinical trials, failed in clinical settings [7–10]. These data indicate that there are significant weaknesses in the experimental models used for the preclinical vaccine testing.

Mice are genetically/immunogenetically well-characterized (compared to other animal models) and are inexpensive and easy to breed, making them a convenient platform for large scale preclinical testing of the effects of various vaccination strategies on tumor growth and metastatic activity. There currently exist several experimental mouse models of HPV infection and associated cancer. Few immunocompetent strains of mice and immunocompromised mice can be infected with Mus musculus papillomavirus 1 (MmuPV1) [11]. While murine papilloma virus based models are very helpful for studying HPV infection, transmission and pathogenesis [12], they are of limited use for testing the vaccine/immunotherapy candidates due to restrictions in the susceptible mouse strains and the requirement for immunogens based on the murine, not human antigens. This limits direct application of MmuPV1-based models for testing human vaccines. Alternatively, whole mouse, or a tissue within a mouse, or murine cells can be made to express HPV proteins, specifically the key oncoproteins E5, E6, and E7 [13]. Examples of these models are: mice transgenic for the early genomic region of HPV16 under the control of the K14 promoter (K14-HPV16) [14–16]; mice xenografted with patient-derived HR HPV expressing tissues/tumor cells [17]; mice *in vivo* transfected with HR HPV plus oncogene(s), such as *Akt* and *c-Myc*, and transposase genes, with subsequent generation of tumors which can further serve as a source of HR HPV expressing tumor cells [18, 19]; mice implanted with syngeneic cells (cell lines) expressing HR HPV oncoproteins inducing formation of tumors and metastases [20–24]. Each of these models has multiple limitations. Transgenic mice develop tolerance to the product of transgene [25], while mice xenografted with patient derived tumors (tumor cells) must be immunodeficient [26], making both models inapplicable for testing of vaccine candidates and/or immunotherapies. Models involving an *in vivo* transfection with HR HPV, human oncoprotein and transposase genes (as [18]) are difficult to reproduce, specifically in large scale vaccine testing experiments.

This leaves the option of murine cells (cell lines) expressing one or more HR HPV oncoproteins, capable of inducing tumor growth in syngeneic mice. These models have several advantages. Firstly, they are based on the common mouse strains inexpensive to maintain, in contrast to transgenic or immunosuppressed animals. Secondly, these mice retain an intact immune system. Thirdly, cell lines can be easily made to express target viral oncoproteins in any desired combination at any level, allowing the modelling of different stages of viral infection and subsequent carcinogenesis [27].

Common targets for therapeutic HR HPV/HPV16 targeting vaccines are the key HPV16 proteins involved in malignant transformation, namely, oncoproteins E6 and E7. Both can elicit cell-mediated responses which could eliminate cells expressing these proteins [28]. A model to test E6/E7-based HPV16 vaccines should, therefore, express either E6, or E7, or both.

The most widely used model of this type is based on the cell lines TC-1 and a clone of TC-1 expressing recombinant luciferase TC-1/luc2 [29]. TC-1 cells were generated from the primary epithelial cells of C57BL/6 mice transformed by expression of HPV16 E6/E7 and c-Ha-ras oncogene [23, 29]. A characteristic feature of these cells was reported to be the high level of expression of *E6* and *E7* mRNA [21]. However, the expression of E6 protein in TC-1 cells cannot be detected. Samorski R *et al.* found TC-1 cells to be totally devoid of any detectable E6 protein [30]. Inhibition of the proteasomal function by lactacystin demonstrated the intracellular accumulation of E6 protein in cells transfected with *E6*, but not in TC-1 cells [30] indicating that in TC-1 E6 was expressed below the detection levels. This contrasts with HPV-associated cervical lesions and cervical cancer, which are characterized by high levels of expression of E6 mRNA as well as E6 protein, both parameters strongly associated with the viral load and the severity of the cervical lesions [31–36].

Secondly, TC-1 cells have not been genetically characterized. Genetic stability/genetic diversification of the cells under longitudinal culture, localization of the inserts of E6/E7 DNA, and stability of E6/E7 expression are basically unknown. Of note, M. Smahel *et al.* demonstrated that after the *in vivo* propagation, TC-1 (sub)clones show changes in morphology, in profiles of expression of a number of genes, such as the monocyte chemoattractant protein 1 (*MCP-1)*, osteopontin (*OSP*), midkine (*MDK*), and *E7* [37]. Furthermore, they exhibit mutations in the sequence of *E7* gene, leading to immune escape [37].

Thirdly, TC-1 cells/tumors exhibit low metastatic activity with metastasis occurring only after intravenous introduction of the cells into mice [38], while tumors grow only after a subcutaneous implantation (see, for example, [39]), and the capacity of TC-1 cells for invasion from the primary tumors is limited [40]. This feature prevents simultaneous testing of the effect(s) of a vaccine or immunotherapy on the growth of solid tumors and their metastatic activity in one experiment, in the same animals.

We aimed to create a new murine model to use in the assessment of therapeutic vaccines against E6 and E7 oncoproteins of HPV16 which would not have these limitations. We based our model on the spontaneously formed murine mammary gland adenocarcinoma 4T1 cells syngeneic to BALB/c mice, namely 4T1 clones expressing luciferase, which we engineered to express HPV16 E6 and E7. The resulting cells were used in challenge experiments to determine if DNA-immunization with HPV16 E6 and E7 can hinder or prohibit their growth and metastatic activity in the prophylactic and therapeutic settings, comparing the results with that obtained in the TC-1/luc2 model.

### 2. MATERIALS AND METHODS

## 2.1 Design of 4T1luc2 cells expressing E6/E7 of HPV16

## 2.1.1 Sequencing of HPV16 E6 and E7 genes

Sequences of *E6* and *E7* genes were retrieved from a retrospective study estimating prevalence of HR HPVs in the cervical tissues of women with high grade cervical lesions and cervical carcinomas diagnosed at the Paul Stradins University hospital in the period 2016–2023. The study was performed according to the ethical permit issued by the Ethical Committee of the Riga Stradins University N2-PĒK-4/415/2022 dated September 26, 2022. Informed consent to use biological materials in research was given by patients at the time of sampling/before treatment.

Formaldehyde fixed paraffin embedded (FFPE blocks) of patients diagnosed with cervical cancer were sectioned into 5 μm thick slices using a microtome (Reichert, Inc., Depew, NY). Sections (10 to 15) collected with sterile forceps were placed into sterile 1.5-2.0 ml Eppendorf tubes and submerged into 400 to 500 μl of sterile mineral oil for PCR. Samples were heated at 90°C for 10 min, and mineral oil containing dissolved paraffin was removed using sterile micropipette. DNA was isolated from the deparaffinized sections using AllPrep DNA/RNA FFPE Kit (Qiagen, Hilden, Germany) according to the manufacturer's recommendations.

Aliquots of DNA (n = 64) were subjected to analysis for HR HPVs using semiquantitative PCR detecting 14 HR HPV genotypes (Anyplex™ II HR HPV Detection kit, Seegene, Seoul, Republic of South Korea) as described by the manufacturer. DNA from the samples positive for HPV16 with high virus load (+++ according to Anyplex™ II, equivalent to Ct < 31 [41]) was subjected to Sanger sequencing of the E6/E7 encoding region. The resulting sequences were deposited in the GenBank with accession numbers PQ215484 to PQ215514. Respective amino acid sequences obtained by translation were aligned using as a reference the sequence of HPV16 isolate NC_001526.4, and the consensus amino acid sequences of HPV16 E6 and E7 were generated using CLUSTAL MUSCLE algorithm of CLC Sequence Viewer 8.0 program (Qiagen, Aarhus, Denmark) (Alignment FASTA files Supl. Fig. S1). The consensus amino acid sequences of HPV16 E6 and E7 were further used to design respective DNA-immunogens.

#### *2.1.2 Generation of lentiviral vector pLJM1_pGK_E6E7 encoding HPV16* E6 and E7

Lentiviral vector pLJM1-EGFP [https://www.addgene.org/19319/] was modified by replacing the sequence encoding EGFP, with the sequence encoding HPV16 E6/E7 from the plasmid carrying full-length genome of HPV16 (pHPV16; www.atcc.org/products/45113d). The immediate early cytomegalovirus (IE CMV) promoter was exchanged for the human EF1-alpha promoter. The latter provides and maintains high transgene expression, transgene stability and copy number [42], and also contains the Sp1 element [43] recognized and regulated by HPV16 E6 and E7 [44]. The E6/E7 coding sequence was preceded by the Kozak sequence to secure the initiation of E6 translation. DNA sequence determining the re-initiation of the E7 translation was preserved. The presence of DNA inserts was confirmed by sequencing. The resulting bicystronic lentiviral vector pLJM1_pGK_E6E7 (Suppl. Figure S2) retained the puromycin-N-acetyltransferase gene under the control of the human PGK gene promoter.

#### 2.1.3 Murine cells stably expressing HPV16 E6 and E7

Cell line TC-1/luc2 (Transformant: Human papillomavirus type 16 (HPV16)

NCBI_TaxID; 333760) [45] was kindly provided by Prof T.C. Wu (John Hopkins University, USA, Baltimore, MD).

Murine mammary gland adenocarcinoma 4T1luc2 cells ("Bioware Ultra Cell Line 4T1luc2," Caliper, Hopkinton, MA, USA; http://www.caliperls.com/assets/014/7158.pdf) were transduced with pLJM1_pGK_E6E7 at a multiplicity of infection (MOI) of 20 transducing units per cell. Individual subclones carrying inserts of E6E7 DNA were obtained by single cell dilution. E6/E7 DNA inserts in the genomes of subclones of 4T1luc2 transduced with E6/E7 lentivirus pLJM-E6E7 were detected by PCR using primer pairs specific to the lentiviral vector (Suppl. Table S1).

### 2.2. Isolation and purification of DNA from murine cells and tissues

DNA was isolated from murine cells using the MagNA Pure 96 DNA Large Volume Kit by MagNA Pure 96 Instrument (both Roche, Basel, Switzerland). DNA from tumors and organs of mice preserved in FFPE blocks was isolated as described for FFPE blocks of human tumors (Section 2.1).

### 2.3 Detection of E6 and E7 DNA and RNA by PCR

#### 2.3.1 PCR assays for detection and quantification of DNA encoding E6/E7

Calibration samples were made of plasmid pLJM1_pGK_E6E7 DNA diluted from $10^6$ to 1 copies in 10-fold serial steps. The number of plasmid copies in the sample was calculated as: DNA amount (ng) x $6.022 \times 10^{23}$ / length of DNA (bp) x $1 \times 10^9$ ng/ml x 650 Daltons [46]. Serial pLJM1_pGK_E6E7 dilutions were analyzed by HPV HR assay Anyplex™ HPV14 allowing semiquantitative, Allplex™ HPV HR (both Seegene) allowing quantitative detection of DNA

encoding HPV16 DNA, both performed as recommended by the manufacturer, and by the in-house PCR test for E6 DNA using primer pair specific to the 5'-end of *E6* (Suppl. Table S1).

In-house PCR was performed in a 25 µl mixture containing 100 ng of DNA, 0.5 µl of primers (10µM) and 2.5 unit Hot Start Taq DNA Polymerase (New England Biolabs Ipswich, MA, USA) on the CFX Connect (Bio Rad, Hercules, CA, USA) or Rotor-Gene Q (Qiagen, Hilden, Germany) instruments using the standard cycling: 95ºC for 5 min, 40 cycles containing 95ºC for 30 sec, Tm of the primer pair for 20 sec, 72ºC for 15 sec.

Thereafter, calibration curves were built attributing the threshold cycle (Ct) in the in-house PCR and in Allplex™ HPV HR to the number of copies of pLJM1_pGK_E6E7 plasmid (Suppl Figure S3). In-house PCR detected up-to 10 copies (Ct values below 32), semiquantitative AnyPlex II HPV HR Detection assay, 1 to 10 copies, and AllPlex HPV HR Detection assay, up to a single copy of E6-encoding DNA (Suppl Figure S3, Panels A-C).

## 2.3.2 Detection of E6/E7 DNA in cells and FFPE tissues

E6/E7 DNA inserts in the genomes of subclones of 4T1luc2 transduced with the E6/E7 encoding lentivirus pLJM-E6E7 were detected by PCR using pair of primers specific to the lentiviral vector (Suppl. Table S1) following the PCR protocol described in Section 2.3.1. The number of copies of E6/E7-encoding DNA in the total DNA extracted from TC-1/luc2 cells and mouse FFPE tissues was determined by AllPlex HPV HR Detection assays (Seegene), or calculated from their respective Ct (cycle threshold) generated in in-house PCR with primers specific to the 5'-end of *E6* gene, using the linear equation based on the calibration curve obtained by in-house PCR of pLJM-E6E7 (see Section 2.3.1 for details).

Presence of the E6/E7 coding sequences in DNA extracted from the FFPE blocks was confirmed by the blinded tests done by the independent operators using semiquantitative Anyplex™ II HPV HR Detection kits (Seegene).

DNA extracted from TC-1/luc2 cells was used as positive control in all assays. No template control was included in all experiments to test for contaminating DNA and as a general control for extraneous nucleic acid contamination, and as a control for primer-dimer formation. The internal control in the in-house PCR assay used primers specific to mouse beta actin gene (*Actb*) (Suppl. Table S1).

#### 2.3.3 Determination of the number of inserts of E6/E7 encoding DNA in the genome of 4T1luc2 subclones by digital drop PCR

The number of genomic *E6/E7* DNA in 4T1luc2_E6E7 B2 and H6 subclones, with respect to the invariant reference loci *Gapdh* and *Actb*, was determined by the digital drop PCR (ddPCR), performed as described [47] with primers specific for *E6*, *E7*, *Gapdh* and *Actb* (Suppl Table S1). Droplets were generated using automated droplet generator (Bio-Rad, Feldkirchen, Germany). Thermocycling was performed on a C1000 Touch Thermal Cycler (Bio-Rad). Data were collected using a QX200 Droplet Reader, and analyzed using QuantaSoft software version 1.7.4.0917 (both Bio-Rad). The results of the primer validation are shown in Suppl Fig S4 (panels A, B). Two clearly distinguishable clusters of positive and negative droplets were observed for *Actb*, *Gapdh*, *E6* and *E7*. No significant amplification was observed for any primer pair in the absence of the template. The copy number of *E6* and *E7* inserts was calculated as the number of detected E6/E7 loci in DNA sample, divided by the number of *Gapdh* and *Actb* loci and multiplied by 4 equivalent to the number of *Gapdh* and *Actb* copies in 4T1 cells [48]. Calculations yielded one copy of *E6/E7* DNA per genome for both subclones (Suppl Fig S4, panels C-F).

## 2.3.4 Isolation of RNA and detection of E6 and E7 mRNA by RT PCR

Total RNA was extracted from the cells and homogenized tissues using GeneJET RNA Purification Kit (Thermo Fischer Scientific Inc.) according to the supplier's protocol. Immediately after preparation, the RNA samples were treated with DNase I (Thermo Fischer Scientific Inc), free of RNase activity, to remove genomic DNA. For this, 1 µl of DNase I (1 unit/µl) was added to 1 µg of RNA, and the mixture was incubated for 30 min at $37^0$C. After incubation, deactivation was performed by adding 1 µL of 50 mM EDTA and incubating the mixture for 10 min at 65°C. RNA was purified from the reaction mixture after treatment with DNase I using the CleanRNA Standard kit (Bio-Connect B.V., Te Huissen, The Netherlands). The RNA concentration was determined spectrophotometrically (DS-11, DeNovix, USA).

First-strand cDNA was synthesized using 1–3 µg of RNA and MMLV reverse transcription kit (Bio-Connect B.V.) according to the supplier's protocol. Q-PCR reactions to detect total *E6*, E6 isoforms *E6*I*, *E6*II*, E6 full-length (*E6FL*), *E7* and mouse *Hprt1* were performed in a 25 µl mixture containing 1 µl (50–100 ng) of cDNA, specific primers (10 µM) (Suppl. Table S1, [49–51]) and 5X qPCRmix-HS SYBR Master Mix (Bio-Connect B.V.) in the Rotor-Gene Q thermocycler (Qiagen). A minus reverse transcription control (-RT control) and no template control were included in RT-qPCR experiments to test for contaminating DNA and as a general control for extraneous nucleic acid contamination and control for primer dimer formation. All measurements were performed in triplicate. RT-qPCR reactions were further confirmed by gel electrophoresis on 2% agarose. Relative gene expression levels were calculated using the ddCt method [52].

## 2.4 Animal Experiments. Ethics Statement

Experiments in laboratory mice were carried out in compliance with the bioethical principles adopted by the European Convention for the Protection of Vertebrate Animals Used for Experimental and Other Scientific Purposes (Strasbourg, 1986). Experimental procedures were approved by the Ethical Committee of the Latvian Food and Veterinary service, permit No 99 valid June 1, 2018 – May 25, 2023. Mice, BALB/c Ola Hsd (H2-Dd) mice (females, 8 weeks old) and C57Bl/6 Ola Hsd mice (females, 8 weeks old) were purchased from Envigo (Venray, The Netherlands) and housed in laboratory animal core facility at the Latvian Biomedical Research and Study Centre (Riga, Latvia). All mice were housed in environment-enriched cages with 3–5 animals per cage at a temperature of 22°C under a 12 h/12 h light-dark cycle with ad libitum access to sterile water and food. Animals were acclimatized for two weeks before starting the experiments. Mouse discomfort during injection of cells or plasmid DNA, or electroporation and monitoring was relieved by inhalation anesthesia as described in [53]. At the experimental end-point, mice were humanely euthanized by cervical dislocation.

## 2.5 Challenge of mice with tumor cells

## 2.5.1 Implantation of tumor cells

Hair on the back was removed with a trimmer and the skin was treated with a depilatory cream (Veet®; https://animalcare.jhu.edu/wp-content/uploads/sites/5/Hair-Removal-on-Rodents-1.pdf ). Cell suspensions to be injected in the amounts indicated in Table 1 were prepared as described previously [54]. Cells were dispersed in 50 µl of complete RPMI and injected subcutaneously using insulin syringes equipped with a 25G needle. Each cell dose was delivered using an individual syringe.

BALB/c mice were implanted with B2 or H6, or parental 4T1luc2 cells (Series I, IV, V, Table 1). In Series I (naïve mice) and V (therapy), tumor cells (10 000) were implanted as single injections to the left from the back of the tail. In Series V (Table 1), implantations were followed on days 3 and 6 by DNA immunization (Section 2.6). Immunization sites were placed at least one cm apart from the sites of implantation. In Series IV (prophylactics), tumor cells were implanted on day 12 post booster immunization as two injections of 5000 tumor cells

Table 1
Experiments in laboratory mice (N/A – not applicable).

| Series NN Mouse strain/ Purpose of experiment | Group | Nn Mice | Immunization/ Injection sites | Tumor challenge | | | |
|---|---|---|---|---|---|---|---|
| | | | | Yes/No | Clone/sub-clone | Dose, cells per injection | Number of injections |
| **Series I.** BALB/C/Tumor growth in naïve mice | I.1 | 5 | N/A | Yes | H6 | 10000 | 1 |
| | I.2 | 5 | N/A | | B2 | 10000 | 1 |
| | I.3 | 3 | N/A | | 4T1luc2 | 10000 | 1 |
| **Series II.** C57BL/6 mice/ Assessment of immune response after DNA-immunization | II.1 | 5 | E6 left/E6 right | No | N/A | | |
| | II.2 | 5 | E7 left/E7 right | | | | |
| | II.3 | 5 | E6 left/E7 right | | | | |
| | II.4 | 5 | pVax1 left/ pVax1 right | | | | |
| **Series III.** C57BL/6 mice/ Tumor challenge followed by DNA-immunization | III.1 | 5 | pVaxE6 left/ pVaxE7 right | Yes | TC-1/luc2 | 5000 | 2 |
| | III.2 | 5 | pVaxE6 left/ pVaxE7 right | | TC-1/luc2 | 5000 | 2 |
| | III.3 | 5 | pVaxE6 left/ pVaxE7 right | | TC-1/luc2 | 5000 | 2 |
| | III.4 | 5 | pVax1 left/ pVax1 right | | TC-1/luc2 | 5000 | 2 |
| | III.5 | 5 | PBS left/PBS right | | TC-1/luc2 | 5000 | 2 |
| **Series IV.** BALB/c/ Prophylactic DNA immunization followed by tumor challenge | IV.1a | 4 | E6 left/E6 right | Yes | H6 | 5000 | 2 |
| | IV.1b | 4 | E6 left/E6 right | | B2 | 5000 | 2 |
| | IV.2a | 4 | E7 left/E7 right | | H6 | 5000 | 2 |
| | IV.2b | 4 | pVaxE6 left/ pVaxE6 right | | B2 | 5000 | 2 |
| | IV.3a | 4 | pVaxE6 left/ pVaxE6 right | | H6 | 5000 | 2 |

| | | | | | | | |
|---|---|---|---|---|---|---|---|
| | IV.3b | 4 | pVaxE6 left/ pVaxE6 right | | B2 | 5000 | 2 |
| | IV.4a | 4 | pVax1 left/ pVax1 right | | H6 | 5000 | 2 |
| | IV.4b | 4 | pVax1 left/ pVax1 right | | B2 | 5000 | 2 |
| **Series V.** BALB/c/ Tumor challenge followed by DNA immunization | V.1 | 5 | pVaxE6 left/ pVaxE6 right | Yes | H6 | 10000 | 1 |
| | V.2 | 5 | pVax1 left/ pVax1 right | | H6 | 10000 | 1 |
| | V.3 | 5 | pVaxE6 left/ pVaxE6 right | | B2 | 10000 | 1 |

(10 000 cells in total) to the right and to the left from the back of the tail, each one centimeter above the immunization site (Table 1). C57Bl/6 mice were implanted with TC-1/luc2 cells (5000 of cells per injection, 10 000 in total) in two sites to the right and to the left from the base of the tail. In the therapeutic settings (Series III, Table 1), DNA immunization was performed on days 3 and 6 after the implantation, DNA was injected into the sites placed at least one cm apart from the implantation sites. DNA-immunization technique is described in Section 2.6.

#### 2.5.2 Assessment of tumor growth

Tumor growth was assessed by bioluminescent imaging (BLI) on IVIS Spectrum (PerkinElmer, Waltham, MA, USA) following the procedures recommended by PerkinElmer [https://cancer.wisc.edu/research/wp-content/uploads/2018/02/Living-Image-32-User-Manual_PN125112-8234.pdf]. Monitoring of bioluminescence was done directly after the implantation, and thereafter every 2–3 days until the experimental end-point on day 21. For detection of bioluminescence, mice were intraperitoneally injected with a freshly prepared solution of D-luciferin (Perkin Elmer) following the procedures recommended by PerkinElmer [https://www.perkinelmer.com/images/APP_Protocol_4-7926_tcm137-196954.pdf]. Bioluminescence imaging was performed as described earlier [54]. Bioluminescence data were processed using LivingImage® software version 4.5 (Perkin Elmer). The level of bioluminescent signal emitted from the site of implantation of tumor cells (Region Of Interest, ROI) was assessed as the total photon flux per second (photons/s).

The size of palpable tumor was assessed by the morphometric measurements done every 2–3 days using calipers starting from day 16 post implantation. Tumor volume was calculated using a standard formula for the xenograft volume $V = xy^2/2$ [55]. Experiments were continued until the tumor volume of the first mouse in the experiment reached the volume of 1 $cm^3$ which was set as the experimental endpoint, often by day 21 post implantation.

#### 2.5.3 Ex vivo assessment of organ infiltration by tumor cells using BLI

Harvesting of lungs, spleens, liver and *ex vivo* assessment of their infiltration by tumor cells was performed using BLI as was described previously [54]. Thereafter, organs were washed five times with PBS, and transferred into 4% formaldehyde solution in PBS and used to prepare FFPE blocks.

### 2.5.4 Histochemical assessment of tumors and organs

The post-fixation processing of the organs was performed following standard protocols, using neutral buffered 10% formalin (Sigma-Aldrich) for 2 h, followed by dehydration with alcohol followed by tissue clearing with xylene and completed with 20 min embedding in paraffin wax (Dia Path, Martinengo, Bergamo, Italy) [56]. FFPE blocks were stored at room temperature maintained at 17°C to 22°C and humidity levels below 60%. FFPE blocks were sectioned on a standard rotary microtome (SLEE medical GmbH, Nieder-Olm, Germany) at a thickness of 5 μm. Sections were picked and placed on the surface of the water in the water bath using tweezers, then picked out of the water bath with microscope slides and stored upright in a slide rack. Slides in the slide rack were dried in the oven as recommended [57]. Dried sections mounted on slides were stained with Mayer's hematoxylin solution, then washed, rinsed, and counterstained with Na and K eosin solution. Thereafter, they were dehydrated, washed with 96% ethanol, cleared from the last wax by xylene and covered with cover slips for microscopic evaluation performed as described earlier [58].

Tumor slides were subjected to pathomorphological assessment and tumor grading. Organ slides were used to establish the presence, size and quantity of metastases, as well as number of inflammatory cells (neutrophils) around tumor areas. Morphological evaluation of organs and tumors of each mouse were observed using a light microscope (Nikon eclipse 55i, Nikon Instruments Inc., Melville, NY) equipped with a camera (DeltaPix Inventio 5SIII, DeltaPix,

Smorum, Denmark) in 10 high-power (×100 magnification) microscope fields. The areas of metastasis or tumor free organs were photographed and processed using specialized computer-assisted morphometry Histological NIS-Elements software (Nikon, Tokyo, Japan). Morphological detection of metastases was based on the standard parameters such as cytological atypia, nuclear size and mitotic activity.

## 2.6 DNA-immunization with HPV16 E6 and E7

### 2.6.1 DNA-immunogens encoding HPV16 E6 and E7 oncoproteins

Genes encoding consensus E6 and E7 (Section 2.1) optimized for expression in the mammalian cells were synthesized (Thermo Fisher Scientific Inc., Waltham, MA, USA). Synthetic genes were cloned into pVax1 (Invitrogen, Carlsbad, CA, USA) under the control of IE CMV promoter, using BamHI and EcoRI sites, generating pVaxE6wt and pVaxE7wt, respectively. Plasmids were modified by site-directed mutagenesis to generate amino acid substitutions in the p53-binding sites of E6 (C70G and I135T; [59]) and in the Rb-binding site of E7 (C24G and E26G; [59]). Site-directed mutagenesis done using the Quick Change II Site-Directed Mutagenesis kit (Agilent Technologies, Santa Clara, CA, USA) yielded DNA immunogens pVaxE6 and pVaxE7, dubbed E6 DNA and E7 DNA, respectively. Presence of DNA inserts was confirmed by sequencing. Plasmids were purified using endotoxin-free DNA purification kits (Qiagen, Germantown, MD, USA).

### 2.6.2 Assessment of expression of E6 and E7 mRNA in eukaryotic cells transiently transfected with DNA-immunogens

4T1luc2 cells were seeded into 6-well plates in the amount of $2 \times 10^5$ cells/well. The next day, cells were transfected with 2 ug of pVaxE6 or pVaxE7 using 5 μL Lipofectamine LTX and 2 μL Plus Reagent (both Invitrogen, Thermo Fisher Scientific Inc). Two days after transfection, cells were harvested to extract total RNA. Total RNA was extracted and assessed by RT-Q-PCR for the expression of *E7* mRNA and total *E6* mRNA (using pair of primers allowing to detect all *E6* isoforms; Suppl. Table S1) (see section 2.3.4 for description).

### 2.6.3 DNA-immunization of mice

Groups of mice were immunized with E6 or E7 DNA-immunogens. Control mice received empty vector pVax1, or PBS (Table 1, Series II). Mice received 20 μg plasmid DNA dissolved in PBS per injection site, delivered intradermally using a 29G syringe. In Series II assessing immunogenicity, in therapeutic Series III and in prophylactic Series IV, injections were delivered in two sites to the left and to the right for from the back of the tail. In therapeutic Series V, injections were delivered in two sites on the right from the back of the tail (one above the other) spaced at least 1 cm apart, to separate the sites of injection from the site of implantation of tumor cells on the left side. All injections were followed by electroporation (EP) performed using the CUY21EditII instrument with the fork-plate electrodes (BEX Ltd., Tokyo, Japan) following protocol described previously [54].

### 2.6.4 Assessment of T cell immune response against E6 and E7

### 2.6.4.1 Synthetic E6- and E7-derived peptides used to test specific immune response

T-cell response in the immunized mice was evaluated using a panel of synthetic peptides (SynPeptide Inc, Shanghai, China). Peptides encompassed the main epitopic regions of HPV 16 E6 and E7 according to IEDB [https://www.iedb.org] (Table 2).

Table 2
Synthetic peptides derived from HPV16 E6 and E7 used to test anti-HPV16 E6 and E7 immune response

| E6 | Position, aa | Abbreviated name | Amino acid sequence |
|---|---|---|---|
| | 1–30 | E6_1.1(R15) | MHQKRTAMFQDPQERPRKLPQLCTELQTTI |
| | 1–30 | E6_1.2(G15) | MHQKRTAMFQDPQEGPRKLPQLCTELQTTI |
| | 26–58 | E6_2 | LQTTIHDIILECVYCKQQLLRREVYDFAFRDLC |
| | 53–90 | E6_3 | AFRDLCIVYRDGNPYAVCDKCLKFYSKISEYRHY |
| | 86–114 | E6_4.1 (L90) | YCYSLYGTTLEQQYNKPLCDLLIRCINCQ |
| | 86–114 | E6_4.2 (V90) | YCYSVYGTTLEQQYNKPLCDLLIRCINCQ |
| | 110–143 | E6_5 | CINCQPLCPEEKQRHLDKKQRFHNIRGRWTGR |
| | 139–158 | E6_6 | MSWTGRCMSCCRSSRTRRETQL |
| E7 | 1–28 | E7.1 | MHGDTPTLHEYMLDLQPETTDLYCYEQL |
| | 22–45 | E7.2 | LYCYEQLNDSSEEEDEIDGPAGQA |
| | 46–72 | E7.3 | EPDRAHYNIVTFCCKCDSTLRLCVQST |
| | 46–72 | E7.3 | EPDRAHYNIVTFCCKCDSTLRLCVQST |
| | 73–98 | E7.5 | HVDIRTLEDLLMGTLGIVCPICSQKP |
| Peptide pools | | | |
| E6/1 | 1–58 | E6_1 + E6_2 | |
| E6/2 | 53–114 | E6_3 + E6_4.1 + E6_4.2 | |
| E6/3 | 110–158 | E6_5 + E6_6 | |

Stimulation of splenocytes was done with single peptides or peptide pools (Table 2) at a total peptide concentration of 10 μg/ml.

## 2.6.4.2 Flow cytometry

In experiments of Series II assessing plasmid immunogenicity, spleens were excised at the experimental endpoint on day 12 after the booster injection, and homogenized. Single cell cultures were prepared using nylon 70 μm cell strainers (Nunc, Roskilde, Denmark). ACK Lysing Buffer (Thermo Fisher) was used to remove the erythrocytes. Evaluation of the immune response was performed using standard protocols [60]. Splenocytes were stimulated with E6/E7-derived peptides at 10 μg/ml for single peptides or a total of 10 μg/ml for all pooled peptides (Table 2) or positive controls, containing phorbol myristate acetate (PMA) at 50 ng/ml and Ionomicin (both from Sigma, Darmstadt, Germany) at 1 μg/ml, or the medium alone. Surface staining of stimulated cells was performed by incubating with FITC-conjugated anti-mouse CD8a (#553031), APC-H7-conjugated anti-mouse CD4 (#560181), and PerCP-conjugated anti-mouse CD3 (#553067) antibodies (all from BD Biosciences Pharmingen, San Diego, CA, USA). Intracellular staining of permeabilized cells was performed with PE-conjugated anti-IFN-γ (#554412), BV421-conjugated anti-IL-2 (#562969) and BV510-conjugated anti-TNF-α (#563386) antibodies specific to murine cytokines (all from BD Biosciences Horizon, San Diego, CA, USA). Samples were analyzed on a FACSAria II cytometer (BD Biosciences, Franklin Lakes, NJ, USA) using FACSDiva software (BD Biosciences). Data were exported as FCS3.0

files and analyzed in FlowJo software (Tree Star Inc, Ashland, OR, USA). The viable lymphocyte population was identified by the lack of FSV660 staining and further sorted by the expression of CD3, CD4 and CD8 surface markers, and the production of IFN-γ, IL-2 and TNF-α.

Immune reactivity of mice was presented as % of CD4 + and CD8 + T-cells producing IFN-γ, or IL-2, or TNF-α, and all three cytokines IFN-γ/IL-2/TNF-α after *in vitro* stimulation with immunogen-specific peptides, E6 peptides for E6 DNA immunized mice, and E7 peptides for E7 DNA immunized mice. Secretion of cytokines in response to stimulation with nonspecific peptides, E7 peptides for mice immunized with E6, and E6 peptides for mice immunized with E7, as well as secretion of cytokines by pVax1-immunized mice in response to stimulation with E6 and E7 peptides served as a control. The percent of CD4 + and CD8 + T-cells secreting cytokines in response to stimulation with individual peptides from E6 (E6_1.1, E6_1.2, E6_4.1, E6_4.2; Table 3) was determined for the pooled splenocytes of mice of each group.

### 2.7 Statistical Analysis

Graphic presentation of the data and statistical calculations were performed using Microsoft Excel, GraphPad Prism version 9.0.2 software (GraphPad Software, San Diego, CA, USA), Statistica 13.5.0.17 software (TIBCO Software Inc. Palo Alto, CA, USA). Nonparametric F-test and Kruskal–Wallis and Mann–Whitney U-tests. A statistical significance threshold of 0.05 was used throughout the study.

### 3. RESULTS

Malignant epithelial cells expressing HR HPV oncoproteins, arising as a result of chronic infection with HR HPVs, specifically HPV16, are characterized by (i) moderate to high levels of expression of E6 and E7 oncoproteins; (ii) the capacity to form E6/E7-expressing solid tumors at the site of ectopic implantation; (iii) the ability of tumor cells to migrate from primary tumors to distal organs; and (iv) the capacity of infiltrating cells to form metastases in distal organs, specifically in the lungs. These features are not fully met in the currently used models, specifically TC-1. Therefore, we aimed to design a new model possessing the necessary characteristics, and allowing an accurate stringent evaluation of the efficacy of therapeutic vaccines targeting neoplasia and tumors associated with HPV16 infection.

#### 3.1 Design of new murine model to test vaccines against chronic HPV16 infection

As a platform, cells/cell line in which to express HPV16 oncoproteins, we selected murine mammary gland adenocarcinoma 4T1 cells, syngeneic to BALB/c mouse strain. 4T1 cells form solid tumors after orthotopic and ectopic implantation, 4T1 tumors form metastases in multiple distal organs and tissues [61–63]. A clone of 4T1 cells expressing luciferase (4T1luc2; https://www.atcc.org/products/crl-2539-luc2) allows for follow up of tumor growth *in vivo*. We have shown that these cells can longitudinally express diverse foreign antigens with little effect on the propensity of the cells to form tumors and metastases [60,64,65].

Although E6 and E7 of HPV16 are highly conserved, slow viral evolution is observed [6,66]. E6 and E7 vaccine antigens could require an update to meet current needs of the therapeutic HPV vaccine development. To check if this is the case, and to choose the sequence of HPV16 E6 and E7 oncoproteins which 4Tluc2 cells would express, we sequenced the genes encoding HPV16 E6 and E7 oncoproteins from cervical cancer tissues collected in Northeastern Europe (Latvia) in 2016-2023 (Gene Bank entries PQ215484 to PQ215514). Sequences of E6 and E7 were found to be identical to the sequence of the HPV16 reference strain NC_001526.4 (Suppl. Figures S1A, B).

The most frequent single amino acid (aa) substitution observed was L90V in E6 (9/31, 26,5%). Of note, 3 of 9 samples carried DNA encoding both L90 and V90 variants of E6 (PQ215490, PQ215495, PQ215503) (Suppl. Figure 1A). In E7, the only aa substitution observed was D4V in PQ215499 (Suppl. Figure S1B). These data corroborated earlier findings on the HPV16 lineage/sublineage prevailing in Europe with a common amino acid substitution L90V in E6 [67,68]. Sequences of E6 and E7 of the reference HPV16 strain were, therefore, selected.

Subclones of 4T1luc2 cells with genomic insert of E6/E7-encoding DNA, dubbed 4Tl1uc2_EEB2 (B2) and 4Tl1uc2_EEH6 (H6), were received by lentiviral transduction. According to ddPCR, both subclones carried one genomic insert of E6E7 DNA (Suppl. Figure S4B). Upon generation, the subclones demonstrated significant difference in the levels of expression of total *E6* and *E7* mRNAs, which were approximately five times higher in H6 than in B2 cells (Table 3). In both subclones, the levels of expression of *E6* were 2,5 times higher than that of *E7* (Table 3).

**Table 3.** Expression of the total *E6* and *E7* in the newly generated 4T1luc2 subclones carry inserts of E6/E7 DNA. Levels *E6* and *E7* mRNA were normalized to *Hprt1,* as one of the most sta housekeeping genes in 4T1 tumors and in mouse tissues [69-73]. Data represent an average o least two independent assays + STDV. N/A – not applicable.

| PCR parameter | Cells | | |
|---|---|---|---|
| | 4T1luc2 | 4T1luc2_EEB2 (B2) | 4T1luc2_EEH6 (H6) |
| Ct (*Hprt1*) | 20.65±0.23 | 20.36±0.12 | 20.98±0.04 |
| Ct (*E6*) | N/A | 27.14±0.05 | 25.11±0.08 |
| Ct (*E7*) | N/A | 28.37±0.02 | 26.53±0.01 |
| ΔCt (*E6*) | N/A | 6.78± 0.01 | 4.13± 0.15 |
| ΔCt (*E7*) | N/A | 8.01± 0.11 | 5.55± 0.25 |

Ratio of the levels of expression of the total *E6* and *E7* mRNA

| | | | |
|---|---|---|---|
| $2^{-\Delta Ct}$ (*E6*) | N/A | 0.009±6.30[illegible]-05 | 0.057±0.006 |
| $2^{-\Delta Ct}$ (*E7*) | N/A | 0.004±2.96E-04 | 0.021±0.004 |
| $2^{-\Delta Ct}$ (*E6*)/ $2^{-\Delta Ct}$ (*E7*) | N/A | 2.349±0.162 | 2.680±0.186 |

#### 3.2 4T1luc2 subclones B2 and H6 longitudinally express E7 and a variety of E6 isoforms

Although the human EF1-alpha promoter directs high transgene expression and ensures maintenance of transgene stability and copy number [42], in mice it is prone to epigenetic silencing by methylation [70]. Furthermore, both E6 and E7 modulate the activity of DNA methyltransferase DNMT1 [71] and can potentially enhance the effect. At the same time, the EF1-alfa promoter contains an Sp1 element [43], which was shown to be bound and activated by E6 and E7 (shown for the Sp1 element in TGF-beta1 promoter; [44]). Hence, we hypothesized that the use of Sp1-containing promoter would secure high levels of expression of both E6 and E7 bypassing the silencing.

To determine, if EF1-alpha promoter and E6 and E7 expression are silenced with time, we compared the levels of *E6* and *E7* expression in cell passages 10 and 40, dubbed here "early" and "late", respectively, using *Hprt1* as a reference housekeeping gene [69]. *Hprt1* functions well in both normal and cancerous cells [72] [73] 74]. The levels of expression of both *E6* and *E7* during the longitudinal passaging increased many-fold (Figure 1A). The effect pointed at no silencing, but, on contrary, an increase of expression from the human EF1-alpha promoter. Expression of *E6* and *E7* in TC-1/luc2 was assessed at a single time point accounted as "late" (cells were kindly gifted by Prof TC Wu in 2019 and cultured, with interruptions, for at least 40 cycles since then). In the late passages, the level of expression of *E6* in TC-1 cells was 3 to 20 times lower, and of *E7*, 8 to 16 times lower than in B2 and H6, respectively (Suppl Table S2).

Cells in the pre-malignant lesions and HPV-related cancers express multiple E6 isoforms, such as the constitutive ones *E6*I*, *E6*II* and the full-length E6 (*E6FL*), and also *E6*III* and *E6^E7* [75–78]. We assessed the capacity of B2 and H6 subclones to longitudinally express the major three: *E6*I*, *E6*II* and *E6FL*. After 40 passages, B2 and H6 still expressed three main E6 isoforms; relative expression levels of *E6*I* and *E6*II* decreased, while *E6FL* increased with time (Figure 1B for B2, and Figure 1C for H6).

This changed the ratio of E6 isoforms in the expressing cells. In the early passages, *E6*I* predominated in both B2 and H6 (Figure 1D). The late passages of B2 demonstrated an increased content of *E6FL* transcript (approaching the levels of *E6*I*), while H6 still demonstrated dominance of *E6*I* over *E6FL* and *E6*II* (Figure 1E). In TC-1/luc2 assessed at a single time point accounted as "late", the *E6F*L transcript was found to be predominant (Figure 1F). Thus, *in vitro*, the late passages of both B2 and H6 showed high levels of expression of *E6* and *E7* mRNA, *E6*I* isoform predominated, furthermore, *E6* and *E7* expression levels increasing with time. All three features mimicked the HPV16-associated cancers.

#### 3.3 Subclones of 4Tluc2 cells expressing HPV16 E6/E7 form solid tumors

To test if B2 and H6 cells can form solid tumors, they were subcutaneously implanted into BALB/c mice in the dose of $1\times10^4$ cells per site, optimal for 4T1 subclones [54]. Solid tumors were generated in all implanted animals. Morphologically, tumors formed by both B2 and H6 cells, represented poorly differentiated adenocarcinomas of solid structure with poor lymphoid infiltration, with small foci of necrosis and in-growth into the muscle tissues, undistinguishable from the tumors formed by the parental cells (Figure 2A-C). Subclones did not differ from the parental cells in the rate of tumor growth (by total photon flux) (Figure 2D). At the experimental end-point tumors tended to be smaller than tumors formed by 4T1luc2, but the difference was not statistically significant (Figure 2E).

Both B2 and H6 tumors demonstrated stable expression of *E6* (total) and *E7* mRNA: ΔCt E6=9.64 and ΔCt E7=9.63, respectively, for RNA extracted from B2; ΔCt E6=7.96 and ΔCt E7= 8.07, respectively, for RNA extracted from H6 tumors (Series I, Table 1). The expression levels of *E6* and *E7* mRNA in H6 tumors exceeded the levels exhibited by B2 tumors by three times, reproducing the difference in the expression levels of total *E6* and *E7* mRNAs observed *in vitro* (Table 3, Fig. 1A).

#### 3.4 Subclones of 4Tluc2 cells expressing HPV16 E6/E7 infiltrate into distal organs

We determined if B2 and H6 cells can migrate from solid tumors to distal organs. Two methods were chosen – *ex vivo* bioluminescence organ imaging (BLI) assessing the photon flux, released by luciferase (Luc) expressing tumor cells migrating into the organ [65], and E6/E7-specific PCR of DNA extracted from the mouse organs. In mouse tissues, BLI has been shown to detect single cells [79], and >10 cells are reliably detected [63]. PCR is likewise sensitive. Our experiments on quantification of E6 DNA have shown that PCR has a sensitivity of 1 to 10 HPV16 copies per sample depending on the PCR protocol (Suppl Fig. S3). In our case, the "HPV16 copies" translates into the number of *E6/E7*-containing mouse chromosomes, which approximates the number of cells, since both B2 and H6 carry single inserts of E6/E7 DNA (we have to keep in mind that 4T1 cells are aneuploid [80]). Each of the methods has limitations. Photon flux generated by a Luc reporter expressed by tumor cells may not adequately quantify the number of tumor cells in the organs in the settings of hypoxia. Hypoxia was shown to compromise Luc performance in both tumor cells and in tissues/organs [81,82]. PCR does not have this limitation and would detect tumor cells in both hypoxic and non-hypoxic settings. However, PCR is performed for DNA isolated from selected organ sections, not the whole organ, and would miss tumor cells outside of the sectioned area. Therefore, we characterized the metastatic activity of B2 and H6 subclones using a combination of two methods. An organ was considered as infiltrated by tumor cells if it was found to be positive by at least one of the methods.

The presence of tumor cells by BLI was detected in 6/8 (75%) lungs, 5/8 (62,5%) spleens, and 6/8 (75%) livers. Using only PCR, we detected tumor cells in 3/8 (37.5%) lungs, 1/8 (12,5%) spleens, 3/8 (37.5%) livers (Figure 3, Suppl Fig. S5, panels A, B and C, respectively). In total, infiltration of *E6/E7*-expressing 4T1luc2 cells into distal organs was detected in 100% of the animals (Figure 3). We have further attempted to evaluate the number of tumor cells infiltrating the organs by BLI and by PCR. BLI detects 3-5 luciferase expressing cells in one image, total flux from one cell equals to 6 500 photons [http://www.caliperls.com/assets/014/7158.pdf], but during *in vivo* passaging, the photon flux can decrease up-to four times due to a decrease in the levels of Luc expression [63], indicating that after passaging, one Luc-expressing cell can emit 1500 to 2000 photons. Based on these data, the organs were populated by 3 to 25 tumor cells (Suppl Fig. S5, panels A, B and C).

By performing E6-specific Q-PCRs of DNAs extracted from the sections of lungs, spleens and livers and operating with respective Ct values, we determined (using the calibration curve which plots Ct values against the number of copies of E6-containg DNA; Suppl Figure S3A) that samples of DNA extracted from the mouse organs contained 5 to 10, and in one case 100 chromosomes with *E6/E7*-inserts. In general, PCR data aligned with the data obtained by BLI, although it did not allow us to determine the exact number of tumor cells infiltrating the organ due to the fact that PCR was done on sections, not the whole organ.

Thus, B2 and H6 cells were able to migrate from the sites of implantation/respective solid tumors into the distal organs, found to be populated by multitude of tumor cells. Most importantly, tumor cells were found in the majority of lungs, indicating the formation of lung (micro)metastases.

#### 3.5 Subclones of 4T1luc2 cells expressing E6/E7 form lung metastasis

Next, using histochemistry, we assessed if infiltrating cells can form metastases in the distal organs, choosing lungs, spleens and livers as organs commonly affected by metastases in the 4T1 model.

Lungs of mice implanted with B2 or H6 were characterized by the pronounced inflammatory polymorphocellular infiltration (Figure 4), which predisposes to the formation of lung metastases [83]. We observed foci of metastatic lesions confined to blood vessels (Figure 4). The quantification of lung metastases was not possible due to the dense inflammatory infiltration. Metastases were found in all lungs positive for tumor cells by the combination of BLI and PCR (BLI+/PCR+; Figure 4). In the lungs of mice with a BLI+/PCR- result, no metastases were detected, which may indicate infiltration of the organ by single tumor cells, generating sufficient photon emission to be detected by BLI, but cells being too few/too dispersed in the organ to be detected by PCR or histochemical assays. Of note, we have also histologically confirmed presence of metastases in a single case of discrepancy between BLI and PCR – mouse #3 with B2 tumor, with lungs negative for tumor cells by BLI, but positive by PCR (Figure 4, panel A, mouse 3). PCR positivity was attributed to a single large lung metastasis.  Cancer cells are hypoxic [84]. Cluster of metastases could have created a local hypoxic microenvironment, interfering with luciferase activity in individual cells, and compromising BLI results.

The spleens of all mice looked morphologically normal. Lymphoid follicles of various shapes were clearly expressed. Germinal centers and perifollicular areas were well visualized. The stroma was somewhat edematous. Tissues showed no signs of fibrosis. Metastases were not detected. Furthermore, no visual differences could be seen in the morphology of spleens affected or not by the infiltrating B2 or H6 tumor cells (Suppl. Figure S6, panel A-D). The detection of single tumor cells or micro-metastases was not possible due to morphological features of the spleen.

No pronounced pathological changes were found in the livers. Metastases were not detected. Interestingly, while tissues showed no signs of necrosis, we observed accumulations of epithelioid cells with atypical nuclei (pathological mitoses) diffusely distributed between the hepatocytes (Suppl Figure S6, panels E-H). Observations resembled earlier findings of the focal collections of specialized histiocytes with secretory (not phagocytic) activity, associated with antigenic stimulation [85,86]. Epithelioid cells or histocytes were detected in both mice positive and negative for the presence of B2 or H6 cells by BLI and PCR (Suppl Figure S6, panel E-H), indicating that they were not associated with the infiltrating tumor cells and formation of (micro)metastases. From the point of view of detection of infiltrating tumor cells and/or metastases, the histochemical analysis of livers was uninformative.

Thus, in contrast to TC-1 cells, which metastasize to the lungs only after the intravenous injections, but not subcutaneous implantation [37,38], ectopically (subcutaneously) implanted B2 and H6 cells were able to infiltrate multiple distal organs (lungs, spleens, livers) and form histologically confirmed metastases in the lungs.

While histochemical analysis confirmed the presence of metastases in lungs, it was not informative for either spleens, or livers. With the exception of the case with a large single metastasis (in the lungs of a B2-implanted mouse) detected by immunohistochemical analysis, BLI alone performed as a fast, simple and reliable method to assess infiltration of organs by tumor cells (a prerequisite to the formation of metastases), and was more objective than PCR as PCR assessed only a part of the organ. Based on this, in further experiments involving 4T1luc2E6/E7 challenge, organ screening for infiltration of tumor cells was performed by *ex vivo* organ BLI.

#### 3.6 DNA-immunization with HPV16 E6 and E7

##### *3.6.1 Design of DNA-immunogens for testing new murine model*

DNA-immunogens pVaxE6 (E6 DNA) and pVaxE7 (E7 DNA) comprised synthetic expression-optimized genes for consensus oncoproteins E6 and E7 with abrogated p53-, and Rb-binding, respectively (Suppl Figure S7), cloned into DNA-immunization vector pVax1. The analysis of E6 and E7 expression had been routinely done on the mRNA level using RT-PCR, as the sensitivity of immunoblotting and immunoprecipitation was earlier shown to be insufficient [87]. 4Tluc2 cells, transiently transfected with pVaxE6 and pVaxE7, expressed the respective mRNAs (Suppl. Fig. S8A, B). The level of expression of *E6* mRNA was 40 times higher than that of *E7* (Suppl. Fig. S8C).

To screen anti-E6/E7 immune response induced by DNA-immunization with E6 and E7 DNA, we used a panel of synthetic peptides encompassing clusters of known B and T cell epitopes of HPV16 E6 and E7 extracted from IEDB database(http://www.tools.iedb.org, download dates: July 2, 2019, and October 12, 2022) (Table 2, Suppl Figure S7).

Major E6 transcripts *E6*I* and *E6*II* encode E6*I and E6*II proteins with the N-terminal 48 aa residues identical to that in the full-length E6FL protein [88]. All the major E6 isoforms are apt to induce an immune response against the N-terminus of E6. The region contains a cluster of strong human and mouse CD4+ and CD8+ T-cell epitopes [89–93]. In humans, cellular response to these epitopes is associated with HPV16 clearance [94]. T-cell response to this region in DNA-immunized mice was to be detected using peptide pool, covering aa 1-58 of E6 (Table 2, pool E6/1).

Aa 15-19 at the N-terminus of E6 harbors its first nuclear localization signal (NLS1) RPRK [88]. Many epitopes are constituted by clusters of charged amino acid residues, which is often used in epitope prediction [95]. This prompted us to determine if NLS1 is a part of a T-cell epitope localized in this region. T-cell response against NLS1 could be beneficial, as it would be difficult for HPV16 to escape from due to the necessity to preserve the nuclear localization of E6. To assess this, aa 1-30 of E6 was represented by two peptides - E6_1.1 with R15 (wild-type NLS1) and E6_1.2 with G15 (mutant NLS1) (Table 2, Suppl Figure S7). G15 was chosen since conversion of R to G in the NLS1 disrupts the nuclear localization of E6 and interferes with its functions [88].

We also sought to determine if the immune response would recognize/distinguish the main amino acid substitution observed in E6, namely L90V (Supplementary Figure 1A). Amino acid substitution L90V was earlier shown to change the affinity of binding of the substitution-carrying peptides to a number of MHC class I alleles [96,97]. Women harboring HLA-B*44, HLA-B*51, or HLA-B*57 not recognizing V90 variant, have been shown to exhibit an increased risk of developing cancer compared with controls [97], indicating an involvement of the T-cell response against an epitope at L90V in the control of HPV16-associated carcinogenesis. Immunogenicity of this region in mice was to be assessed using synthetic peptides covering aa 86 to 114 of E6 with L90 (E6_4.1) and with V90 (E6_4.1) (Table 2, Suppl Figure S7).

Peptides were used for *in vitro* stimulation of splenocytes of the DNA-immunized mice either individually (as E6_1 and E6_4 variants, and E7 peptides) or in pools (aa 1-58, 53-114, and 110-158 of E6; Table 2).

#### *3.6.2. Immunogenicity of plasmid DNA* encoding HPV16 E6 and E7

Groups of C57Bl/6 mice were DNA-immunized with E6, E7, or E6/E7, or empty vector twice with three weeks interval (Table 1, Series II) following a protocol which secures strong immune response against a wide panel of viral immunogens [53,54,60]. An interference of co-injected DNA-immunogens was repeatedly described [53,98,99]. The levels of transcription of *E7* were significantly higher than that of *E6* (Suppl Fig. S8C), indicating possible competition of *E7* and *E6* for the transcription machinery. Hence, in E6/E7 group, mice received E6 DNA and E7 DNA as separate injections (left hip and right hip) (Table 2).

The immune response of stimulated splenocytes of immunized mice was assessed by multiparametric flow cytometry with intracellular staining (ICCS) as percentage of CD4+ and CD8+ T cells responding to peptide stimulation by simultaneous production of IFN-γ, IL-2 and TNF-α. The latter quantifies proliferating cells with the highest levels of specific cytokine production [100,101]. We detected anti-E6 immune response against aa 1-58 (pool E6/1), 53-114 (pool E6/2), 110-158 (pool E6/3) by CD4+ (Figure 5A), but not by CD8+ T cells (Figure 5E). DNA-immunization with E7 induced no detectable CD4+, or CD8+ T cell response (Figure 5B, F). DNA-immunization with E6/E7 induced CD4+ T cell immune response against aa 110-158 (pool E6/3) (Figure 5C). No specific responses were observed in the vector-immunized mice (Suppl Figure S9).

Four mice (80%) in E6 DNA group, and two mice (40%) in the E6/E7 DNA group responded to stimulation with at least one pool of E6 peptides (Figure 5, panel I). In E6/E7 DNA group, one mouse (20%) responded to stimulation by both E6 and E7-dervied peptides (Figure 5I). Contrary to our expectations, the N-terminal region was not immunodominant (3/10 mice recognizing each of E6 pools; Table 3; Fig. 5I). No E7-specific responses were observed in mice receiving E7 DNA group (Figure 5I).

Further, using pools of splenocytes from each mouse group, we looked for the presence or absence of immune recognition by CD4+ and CD8+ T cells of the individual E6 peptides, comparing R15 versus G15, and L90 versus V90 peptide variants (Figure 6). Specific secretion of IFN-γ and triple secretion IFN-γ, IL-2 and TNF-α in response to splenocyte stimulation with the peptide E6_1.1 presenting R15 was detected in both CD4+ and CD8+ T-cells of mice immunized with E6 DNA, while E6_1.2 representing G15 was not recognized (Figure 6A, C, D, G). CD4+ T-cells of mice recognized neither peptide E6_4.1 presenting L90, nor peptide E6_4.2, presenting V90, while CD8+ T cells responded by triple IFN-γ/IL-2/TNF-α secretion to both E6_4.1 (L90) and E6_4.2 (V90) (Figure 6 G). Thus, R15 of NLS1 RRPK motive was involved in the recognition of the epitope at aa 1-30 of E6 by both CD4+ and CD8+ T cells. The region 86-114 of E6 contained an epitope of CD8+ T cells, but aa residue 90 was not involved in the immune recognition, at least in C56Bl/6 mice.

##### *3.6.3 Splenocytes of mice DNA-immunized with E6 and E7 demonstrate an activation*

When analyzing cytokine secretion, we noted an increased level of IFN-g, IL-2 and TNF-a production by the cultured unstimulated splenocytes of mice immunized E6 and E7 (Figure 7). Cultured unstimulated CD4+ cells of E6-mice overproduced IFN-g and IL-2 (Figure 7A, B), no effect was observed for CD8+ T cells (Figure 7D, E). Cultured unstimulated CD4+ and CD8+ T cells of E7-immunized mice overproduced TNF-a (Figure 7C, F). No effect was observed in E6/E7-mice which received two-fold lower doses of both plasmids (Table 1, Series II; Figure 7A-D). An increase was not due to the injection of plasmid DNA and/or electroporation, since no increase in % cells secreting IFN-g, IL-2 and/or TNF-a was observed in mice DNA-immunized with vector (Figure 7A-D). These data corroborated earlier findings of E6- and E7- induced immune activation [102–105]. Interestingly, however, while E6 stimulated immune environment characteristic to T-cell activation/induction of Th1 immune response/T-cell memory responses [100], concordant with the induction of specific immune response by CD4+ T cells (Figure 5A, I), E7 induced an inflammation [106] but no E7-specific immune response (Figure 5B, F, I).

#### 3.7 DNA-immunization with E6, but not E7, shows therapeutic effect in the TC-1 model

We further tested the functionality of the induced immune response, i.e. if E6 and/or E7 DNA-immunization can hamper growth and metastatic activity of TC-1/luc2 cells in a therapeutic setting. C57Bl/6 mice were injected with a dose of cells earlier used by T.C. Wu *et al.* in similar experiments [107]. Three and six days after implantation, mice were DNA-immunized with E6, or E7, or E6/E7, control mice received pVax1 or PBS (Table 1, Series III). In lines with results of immunogenicity tests, DNA-immunization with E6 suppressed tumor growth as judged by the size of tumors at the experimental end-point, whereas E7 and combination of E6 and E7 had no effect (Figure 8A).

Infiltration of tumor cells into the lungs, spleen, livers was assessed by *ex vivo* BLI. In all animals the photon flux from the internal organs did not exceeded the background levels (Figure 8A). Livers are not affected by TC1 metastases [37] (TC-1 cells form metastases in the liver after injection of the cells into the anterior mesenteric vein [108]). In our case, photon flux from the lungs did not differ from that from the liver (Figure 8B) confirming that distal organs, including lungs, were not infiltrated by tumor cells.

We performed histochemical screening of the livers to determine if implantation of tumor cells followed DNA-immunization with E6/E7 had any effect on the organ, as C57Bl/6 mice are highly susceptible to liver injury [109]. All mice were found to have immune infiltrates in the liver (Figure 9A), similar to the epithelioid cells (histocytes) observed in the livers of TC-1 implanted naïve mice (see Suppl Figure S6, panel E-H). The average number of immune infiltrates in the groups did not differ (Figure 9B), but their total number (in all screened fields) was higher in the DNA-immunized mice compared to the control group. The total number of infiltrates in mice DNA-immunized with E7 tended to be higher than in mice receiving E6 ($p=0,1$) and was significantly higher than in the E6/E7 and control groups ($p<0,05$) (Figure 9C). Thus, in the TC-1 model, DNA-immunization with E6 reduced tumor growth, while E7 had no effect, as could have been expected from our immunogenicity data and earlier findings (Section 3.6.2). The TC-1 model did not permit assessment of the effect of DNA-immunization on the infiltration of organs by tumor cells. However, it was noted that all groups of TC-1/luc2 challanged C57bl/6 mice demonstrated signs of liver inflammation, which tended to be more pronounced in DNA-immunized mice, specifically in the recipients of E7 DNA.

#### 3.8 DNA-immunization with E6 and E7 partially protects against challenge with E6/E7-expressing 4T1luc2 cells

We next sought to determine the functionality of the immune response induced by DNA-immunization with E6 and E7 using the newly generated E6/E7 expressing 4T1luc2 cells, i.e. if DNA-immunization E6 and E7 can reduce their tumorigenic and metastatic activity in a prophylactic setting. BALB/c mice were DNA-immunized with E6, E7, or E6/E7, or empty vector, and 12 days after the boost were challenged with B2 or H6 subclones in a dose of $5\times10^3$ cells per site (Table 1, Series IV) (same dose in case of TC-1/luc2 cells, Table 1, Series III). Primary tumors developed in all mice. Growth parameters of tumors formed by B2 and by H6 cells did not differ in either E6, or E7, or E6/E7-immunized groups (Suppl. Figure S10). Hence, results obtained for mice challenged with B2 and with H6 cells were pooled. By the end of the follow-up (day 21), a significant difference in tumor weights was observed in mice DNA-immunized with E6 compared to the control group ($p=0,023$) (Figure 10A). DNA-immunization with E7 tended to reduce tumor weights, although the effect did not reach the level of significance ($p=0,062$). No effect was observed for mice DNA-immunized with E6/E7 (Figure 10A).

Total photon flux from the organs (lungs, spleens and livers) for mice challenged with B2 did not differ from that in mice challenged with H6 cells (Suppl. Table S3). Hence, mice were also pooled into one B2/H6-challenged group. Immunization with E6 significantly reduced migration of tumor cells to distal organs, seen as a decrease in the sum of flux from all organs, and, importantly, from the lungs (Figure 10 B). DNA-immunization with E7 and with E6/E7 also caused significant reduction of cell migration into distal organs (lower total flux of all organs; Figure 10 B) and tended to reduce migration of tumor cells into the lungs (Figure 10C; p=0,08 and p=0,06, respectively).

After detecting liver inflammation in the TC-1/luc2 challenged C57bl/6 mice, we sought signs of liver damage in DNA-immunized B2/H6-challenged BALB/c mice. H&E stained sections of livers showed clusters of atypical cells with large basophilic nucleus and basophilic cytoplasm, with inclusions of neutrophils and lymphocytes (immune cell infiltrates) (Figure 11A). Their numbers were increased in mice DNA-immunized with E7 and decreased in mice DNA-immunized with E6, while DNA-immunization with E6/E7 had no effect (Figure 11B).

Thus, DNA-immunization with E6 hindered, and with E7 tended to hinder growth of the E6/E7-expressing 4T1luc2 tumors, and both limited the capacity of tumor cells to migrate into distal organs. Failure of E7 to perform in the TC-1/luc2 challenge in therapeutic settings, and the relatively weak performance in the E6/E7-4T1luc2 model in prophylactic settings, along with involvement in the induction of liver damage prompted us to exclude E7 DNA-immunogen from further testing.

#### 3.9 DNA-immunization with E6, effective in the TC-1/luc2 model, has no therapeutic effect against challenge with E6/E7-expressing 4T1luc2 cells

Next, we assessed if DNA-immunization with E6 can affect tumorigenic and metastatic activity of the newly generated E6/E7 expressing 4T1luc2 cells in a therapeutic setting, reproducing experiments done in TC-1/luc2 cells. BALB/c mice were challenged with *E6/E7*-expressing 4T1luc2 subclones B2 or H6, and 3 and 6 days post implantation DNA-immunized with E6; control mice received empty vector (Table 1, Series V). DNA-immunization with E6 hindered growth of B2 tumors by day 8, but the effect disappeared by day 11 (Figure 12, Panels A, B). Although the suppressive effect was short-term, B2-implanted/E6 DNA-immunized mice tended to have lower tumor weights at the experimental end-point compared to the vector immunized mice (Figure 12C). Immunization had no effect on the tumors formed by H6 cells.

Total photon flux from the distal organs in the B2-implanted mice receiving E6 DNA was lower than in mice receiving empty vector (Figure 12D). No difference was observed for mice implanted with H6 (Figure 12D). E6 DNA-immunization had no effect on the infiltration of B2 and H6 cells into the lungs (Figure 12E).

Randomly selected lungs and spleens, as organs shown by BLI to be infiltrated by B2 and H6 cells in naïve mice, were subjected to the analysis by commercial PCR by an independent operator (Anyplex™ II HR HPV Detection kit, Seegene; detects 1 to 10 copies of E6 DNA, see Suppl. Figure 3C). Organs of mice challenged with H6 and DNA-immunized with E6 tended to exhibit lower frequency of detection of HPV16 E6 DNA than the controls (non-immunized or vector immunized mice; Table 4), although no therapeutic effect of E6 DNA-immunization on these mice was observed in the experiments assessing tumor growth or infiltration of organs by tumor cells. No reduction was observed for the B2-challenged mice DNA-immunized with E6 (Table 4). Thus, in a therapeutic setting, DNA-immunization with E6 exerted little/no effect on either the tumorigenic, or metastatic activity of the *E6/E7*-expressing 4T1luc2 cells, in contrast to more positive performance shown here in the TC-1/luc2 model.

Table 4. Analysis of DNA extracted from a selection of organs of B2 or H6 implanted mice, either immunized (naïve) or subjected to the therapeutic immunization with pVaxE6, or pVax1 (Series V, Ta 1) for the presence of HPV16 DNA using commercial PCR test (Anyplex™ II, Seegene).

| Mice | Challenge cell line | Spleen | | Lungs | | Total tested samples | Total positive samples | % positive samples |
|---|---|---|---|---|---|---|---|---|
| | | nn tested | nn HPV16 DNA+ | nn tested | nn HPV16 DNA+ | | | |
| Naïve | B2 | 2 | 0 | 2 | 0 | 4 | 0 | 0 |
| pVax1 immunized | | 4 | 3 | 4 | 2 | 8 | 5 | 63 |
| Controls (naïve+pVax1) | | 6 | 3 | 6 | 2 | 12 | 5 | 42 |
| pVaxE6 immunized | | 4 | 2 | 5 | 2 | 9 | 4 | 44 |
| Naïve | H6 | 2 | 2 | 2 | 2 | 4 | 4 | 100 |
| pVax1 immunized | | 4 | 2 | 5 | 3 | 9 | 5 | 56 |
| Controls (naïve+pVax1) | | 6 | 4 | 7 | 5 | 13 | 9 | 69 |
| pVaxE6 immunized | | 5 | 1 | 5 | 2 | 10 | 3 | 30(*) |

(*) p=0,069 - E6 immunized mice compared to controls using "N-1" Chi-squared test Campbell (2007) and Richardson (2011) [111,112].

## 4. DISCUSSION

HPV16 is the most prevalent high risk genotype, and currently the main target of the therapeutic vaccines against HR HPV-associated neoplasia and cancer [5, 7]. Over 50% of the therapeutic HPV16 vaccines tested by now target the oncopoteins E6 and E7 [5, 7]. Many of these vaccines were successful in the preclinical testing, but failed in clinical trials [5, 10, 45, 113]. One of the reasons could be the inadequacy of the murine model(s) used in preclinical testing, namely, their limitations in capacity to accurately mimic human disease.

The most commonly used HPV-associated cancer model to test therapeutic vaccines against HPV16-associated cancer are TC-1 cells, epithelial cells from murine lungs completely transformed by the human c-Ha-ras oncogene and HPV16 *E6/E7*, and their luciferase-expressing derivative TC-1/luc2 [23, 29]. The discrepancy between the performance of vaccine candidates in tumor challenge experiments with TC-1 and TC-1/luc2 cells [45, 113] and their clinical performance could be due to the specific limitations of this model, namely difference of these cells from the HPV16-associated tumor cells [31–36]. This drives the need for development of new murine models for testing therapeutic HPV16 vaccines which would reproduce the HPV16-associated cancer cells better than the TC-1 cells.

To design a therapeutic model targeting HPV16 E6 and E7, we chose as the platform the well characterized mammary gland adenocarcinoma 4T1 cells, a highly tumorigenic and invasive transplantable tumor cell line [114]. In contrast to TC-1, 4T1 cells are spontaneously formed tumor cells. The cell line is poorly immunogenic in mice [114], ensuring high tumorigenicity and invasiveness. 4T1 cells metastasize from the primary tumor to the multiple distant sites including lymph nodes, blood, liver, and lungs [114]. The 4T1 cell line is comparatively (genetically) stable with the number of mutations (SNVs and indels) found to be an order of magnitude smaller than in other cancer cell lines [48]. We have based our model on a derivative clone of 4T1 which expresses optimized luciferase (Luc) 4T1luc2, and thus allows for *in vivo* monitoring of tumor growth and metastases formation [63]. We have previously shown that the capacity of these cells to form tumors and to metastasize into distal organs is not affected by the expression of the foreign antigens [54, 60, 65]. Together, the aggressiveness, high metastatic activity, low mutational burden, and tolerance to foreign protein expression, positioned 4T1 (4T1luc2) cells as an ideal platform for expression of foreign proteins/antigens, here the HPV16 E6 and E7 oncoproteins.

Analysis of genes encoding *E6* and *E7* of over 300 HPV16 isolates circulating in Europe together with E6 and E7 sequences obtained in this study, demonstrated high conservation of both oncoproteins, with consensus sequences identical to that of the reference HPV16 strain (NC_001526.4). Hence, we used the sequences of E6 and E7 of the reference HPV16 strain in both the E6/E7 murine model, and for the E6/E7-targeting DNA-immunogens to be used to test this model. In this sense, our model was similar to TC-1 [https://www.cellosaurus.org/CVCL_4699]. Using lentiviral transduction, we generated subclones of 4T1luc2 cells carrying one genomic insert of E6/E7 DNA. We placed the insert under the control of the human elongation factor 1 alpha (EF1-alfa) promoter instead of the original IE CMV promoter contained in the lentivirus, as it provides high levels of transgene expression [42]. Also, it contains an Sp1 element, known to be positively regulated by E6 and E7 [44], which we thought would ensure high levels *E6/E7* expression.

Two subclones of 4T1luc2 cells were selected EEB2 (B2) and EEH6 (H6), exhibiting varying levels of expression of *E6* and *E7* mRNA with H6 expressing approximately twice the level of E6/E7 mRNA compared to B2. The late passages of both B2 and H6 showed a significant increase in *E6* and *E7* expression compared to the early passages. The observed increase was unlikely to result from a decrease in the expression levels of the reference housekeeping gene *Hprt1*, as *Hprt1* is one of the most stably expressed genes in the cells of both healthy and tumor bearing mice [72]. Furthermore, high levels of *Hprt1* expression are characteristic to the aggressive cancers/cancer cells (squamous cell carcinomas which we model, as well as adenocarcinomas as are 4T1 cells; [73]). The levels of *Hprt1* expression increase, not decrease, with tumor aggressiveness/progression (see for example, [74]). Such an increase in the level of *Hprt1* expression would cause a relative decrease in the levels of expression of *E6* and *E7*, which was not the case. With this, an increase in the levels of expression of *E6* and *E7* was considered as an objective event occurring in the E6/E7-expressing 4T1luc2 cells under the longitudinal passaging. The effect indicated that no silencing occurred and confirmed that EF1-alpha promoter was optimal for securing high levels of longitudinal E6 and E7 expression in 4T1luc2 cells.

We compared B2 and H6 with TC-1 cells in the capacity to express *E6* and *E7* mRNA. *E6* expression levels in the late passages of B2 cells were 3 times and of H6 cells, 20 times higher than in TC-1 cells. Also, the expression levels of *E7* were 8 (for B2) to 16 times (for H6) higher than that in TC-1. Both E6 and E7 are immunosuppressive, affecting both innate and adaptive immune responses [115, 116] with a synergistic inhibitory effect [117]. *In vivo*, in mice, E7 reduces certain innate immune cell populations (APCs, namely Langerhans cells (LC)) and suppresses proliferative CD8 T cell responses [118]. TC-1 cells may have yielded better results in the therapeutic immunization performed in this study due to the lower expression levels of E6 and E7 and, hence, a reduced systemic effect on the implanted mice compared with B2 or H6 subclones.

We have also shown that TC-1 cells demonstrate a significant difference from B2 and H6 cells in the abundancy of *E6* isoforms. In pre-malignant lesions and HPV-related cancers, there exist a wide variety of *E6* isoforms generated by alternative splicing of RNA characteristic to the high risk HPVs [76, 119]. The two major splice isoforms are *E6*I* and *E6*II* [120–122]. *E6*I* mRNA supports the translation of E7 oncoprotein (after reinitiation), while un-spliced E6 mRNA supports the expression of the full-length E6 protein and a low level of expression of E7 [119, 123, 124]. The E6*I isoform is a crucial actor in the HPV16-driven carcinogenesis and has important functions distinct from those of the full-length E6 isoform (E6FL) [75, 125–129]. More so, functions of the full-length E6 and E6*I were repeatedly shown to be opposing [87, 125] and could be even “counteracting” [130]. After directly cloning and also after longitudinal passaging, B2 and H6 subclones predominantly expressed *E6*I* mRNA. In contrast, TC-1/luc2 cells predominantly express *E6FL.* Thus, the levels of expression of total E6 and of E7, and the ratio of *E6* isoforms in B2 and H6 subclones, but not in TC-1, reproduced the pattern of oncoprotein expression observed in HPV-associated cancers [131].

Heterogeneity in the levels of expression of E6 transcripts on diverse cell lines are due to differences in cellular factors acting on the splicing sequences of the E6/E7pre-mRNA [132]. The process of alternative splicing is regulated by cis-acting elements and trans-acting factors. Serine and arginine-rich splicing factors (SRSFs) [133] and heterogeneous nuclear ribonucleoproteins (hnRNPs) [78] are two essential auxiliary factors in regulating the usage of splicing sites through the recognition of specific cis-acting RNA elements. HPV16 infection induces overexpression of SRSF1, SRSF2, and SRSF3; the key regulator of HPV16 oncoprotein expression and cervical tumor maintenance is SRSF2 [134]. Interestingly, SFSRs are overexpressed (due to mRNA upregulation and/or DNA amplification) in ≥ 10% of breast cancers [135]. Amplification of *SRSF2* gene, critical for splicing of HPV16 E6/E7pre-mRNA, has been reported in 6–13% of breast cancer cases [136, 137]. Furthermore, *SRSF2*-overexpressing tumors represent > 20–40% of cases of human triple-negative breast cancer (TNBCs), the most aggressive breast cancer subtype [135] modelled by 4T1 cells [138]. Expression of SFSRs, specific to breast cancer cells, namely their propensity to perform HPV16 E6/E7pre-mRNA splicing, may be one of the factors determining the prevalence of E6*I isoform in *E6/E7*-expressing 4T1luc2 cells, making these cells a better platform for the HPV16 murine model than other cell lines used so far.

Therapeutic vaccine testing in the murine HR HPV models involves the evaluation of the capacity of HR HPV immunization to cause rejection of tumors, or at least to hinder their growth, and for the ability to prevent or reduce the formation of metastases. We assessed the tumorigenicity of *E6/E7*-expressing 4T1luc2 cells *in vivo* in the syngeneic immunocompetent BALB/c mice. After ectopic implantation, both B2 and H6 subclones formed palpable tumors within 6–7 days after implantation (at relatively low dose). Tumors reached in size 1 $cm^3$ in size within 3–4 weeks and had a morphology undistinguishable from the parental 4T1 tumors. Stable *E6/E7* expression by B2 and H6 subclones of 4T1luc2 cells was reproduced in tumors which even demonstrated an increase of the levels of *E6/E7* mRNA. Growth parameters were similar to those observed in the TC-1 model [https://td2inc.com/tc-1-syngeneic-model], with both models allowing for the evaluation of the effects of therapeutic vaccination on tumor growth in a one month experiment.

Metastatic disease develops in 15 to 61 percent of women with cervical cancer. A database study by Shu Zhou and Fang Peng has shown that the most common single metastatic site in cervical cancer is the lungs (37.9%) [139]. Frequent lung metastasis had been explained by the “seed and soil” hypothesis according to which lungs present a favorable microenvironment benefiting the survival and proliferation of cervical cancer cells. Lung metastases were long since found to be a correlate of poor prognosis, occurring in 3–5% of CC cases and cervical adenocarcinoma cases [140]. Notably, we found TC-1 cells to be compromised in their ability to migrate to distal organs, specifically lungs when introduced by subcutaneous injections used to generate primary tumors. In contrast, subcutaneously implanted *E6/E7*-expressing 4T1luc2 cells migrated from the primary tumors into multiple distal organs and formed histologically proven metastases in the lungs. With this, *E6/E7*-expressing 4T1luc2 cells offered a possibility to assess the effect of therapeutic immunization on both tumor growth, and on the migration of tumor cells/formation of metastases, which is of great importance for the evaluation of the potential of a therapeutic cervical cancer vaccine.

To test the new challenge model in comparison to TC-1 (TC-1/luc2), we used DNA-immunogens matching those expressed by the murine tumor cells. The immune response was screened using a panel of peptides encompassing the main epitopic regions of E6 and E7. E6 and E7 DNA-immunizations were performed using an optimized immunization protocol working well in both C57Bl/6 and BALB/c mice [141, 142]. E6/E7 DNA-immunized mice received E6 and E7 DNA as separate injections to circumvent the competition for transcription machinery and also the suppression of immune response against E7 due to E6 immunodominance [143]. In the E6 DNA-immunized C57Bl/6 mice, we detected triple IFN-g/IL-2/TNF-a production by CD4 + T cells specific to multiple epitopes throughout the protein, and by CD8 + T cells, to an epitope at the C-terminus of E6. No T-cell response was directed against E7. Our findings were similar to the findings of earlier studies on the strong immune response induced by DNA-immunization with E6 [30, 144–150], but not E7 [145, 151–153] which is immunogenic if fused to signals retargeting intracellular trafficking or antigen processing [151, 152, 154]. Interestingly, DNA-immunization with E6 and E7 delivered at separate sites, demonstrated a reduced anti-E6 immune response compared to immune response in mice receiving only E6. The effect could result from a two-fold lower dose of E6 DNA, or a systemic effect of E7. In this context, it is worth noting that priming of mice with E7 DNA reduced the efficacy of boosting by the viral vector expressing the same antigen [145], pointing at a systemic E7-induced immune suppression.

We have further tested the E6/E7-expressing 4T1luc2 cells as a tool to assess functionality of the immune response induced by DNA-immunization in the prophylactic challenge in BALB/c mice. In concordance with the results of immunogenicity tests, DNA-immunization with E6 reduced the size of tumors at the experimental endpoint. It has also reduced infiltration by tumor cells into distal organs, specifically lungs. DNA-immunization with E7 rendered a weak inhibitory effect which did not reach statistical significance. DNA-immunization with combination of E6 and E7 had no effect on tumor growth, although it tended to reduce organ infiltration by tumor cells. In general, these results were similar to the ones obtained in the TC-1 model in a therapeutic setting.

Notably, we observed that DNA immunization with E6 induced markers of Th1 type of T cell immune response [100], and with E7, markers of inflammation [106]. The latter were initially seen as high levels of TNF-a production by unstimulated splenocytes of E7 DNA recipients and then as pathological changes in the liver, characterized by an increase in the number of clusters of atypical cells/immune infiltrates. Changes in the liver were similar to that observed in response to ConA treatment, used in mice to model hepatic fibrosis and cirrhosis [155–157]. This effect of HPV16 E7 cannot be linked to the Rb-binding capacity as the latter was abrogated by site-directed mutagenesis. Due to poor immunogenic performance and hepatotoxic effects, E7 was removed from further assessment. Testing of the therapeutic effect of DNA-immunization using E6/E7-expressing 4T1luc2 cells was done only for E6.

In a direct comparison of *E6/E7*-expressing 4T1luc2 and TC-1/luc2 cells, the latter demonstrated more positive results of the therapeutic DNA-immunization, as E6 was shown to hinder the growth of TC-1/luc2 tumors. These data align with the earlier studies which have shown that DNA-immunization with expression optimized E6 or its fusions, devoid of p53-binding binding motifs, fully or partially protected mice from TC-1 challenge [30, 144, 146–150]. We could not demonstrate a likewise therapeutic efficacy of E6 DNA-immunization in the *E6/E7*-expressing 4Tluc2 model (only an insignificant reduction of tumor growth for subclone B2, but not H6).

Since we were unable to detect the migration of TC-1 cells in the distal organs, we could not use TC-1 model to assess the effects of DNA-immunization on the metastatic activity of the tumor cells. In contrast, B2 and H6 cells migrated from primary tumors into multiple distal organs and formed histologically proven metastases in lungs. Using E6/E7-expressing 4T1luc2 cells we have demonstrated the potential of DNA-immunization with E6 to hinder migration of tumor cells into the distal organs (as assessed by BLI). The effect was, again, observed for subclone B2, but not H6. The only positive effect of DNA-immunization with E6, observed in mice challenged with H6 subclone, was that fewer organs were PCR-positive for HPV16 (E6) DNA. Interestingly, the phenomenon was not observed in the B2-implanted mice. This could be due to the fact that for a more aggressive H6, more mice/mouse organs were affected by the metastases, allowing us to see the difference between the control mice and mice immunized with E6

DNA. However, tumor cells were detected by PCR only in the selected organ sections, not whole organs. With this, no effect of E6 DNA-immunization on the organ infiltration by tumor cells shown by BLI was a more objective result. It is worth noting here that the effects of E6 DNA-immunization on tumor growth and tumor cell migration were observed for B2 cells, which *in vitro* expresses lower levels of *E7* and *E6*, specifically lower levels of the *E6*I* isoform, and may, therefore, have a weaker systemic effect on the implanted mice (cause less immunosuppression).

Overall, despite measurable positive effects of E6 in a therapeutic setting in the TC-1 model, and a prophylactic setting in the 4T1luc2-based model, the therapeutic challenge of E6 DNA-immunized mice with 4T1luc2 cells expressing E6/E7 produced disappointing results. E6 was unable to restrict growth of the *E6/E7*-expressing tumors and had little effect on the metastatic activity of tumor cells. With this, the *E6/E7*-expressing 4T1luc2 cells worked as a more reliable stringent model than TC-1 cells, permitting assessment of the efficacy of therapeutic immunization with E6 DNA on both primary tumor growth and metastatic activity of tumor cells.

## 5. CONCLUSIONS

E6 DNA expressed from the consensus HPV16 E6 sequence, delivered into C57Bl/6 mice was immunogenic on a T-cell level, while E7 DNA induced no detectable T-cell response. The E6/E7 DNA combination reduced immune response against E6 despite separate E6/E7 delivery. T-cell response against E6 was targeted against multiple epitopes, including one in the NLS1 region. Common amino acid substitution L90V in E6 played no role in the immune recognition of E6 by either CD4+, or CD8 + T-cells. In lines with the results of immunogenicity tests, TC-1/luc2 challenge of C57Bl/6 mice followed by DNA-immunization with E6 and E7 demonstrated partial therapeutic effect of E6, but not of E7, or of the E6/E7 combination. The TC-1 model did not permit assessment of the anti-metastatic potential of the immunogens due to low/no infiltration of tumor cells into distal organs. Despite immunogenicity of E6 DNA for T-cells and its acceptable performance in the therapeutic TC-1/luc2 challenge, it provided very limited/no therapeutic effect in BALB/c mice challenged with *E6E7*-expressing 4Tluc2 cells with regards to tumor growth, final tumor volumes, or infiltration of distal organs by the tumor cells. This demonstrates the utility of the 4T1luc2-based model for stringent testing of E6/E7-based experimental vaccines against HPV16. Our data on the difference in the performance in challenge of tumor cells expressing varying levels of HPV16 oncoproteins and their isoforms demonstrates the importance of these parameters in the model and the need to maximize the consistency of oncoprotein expression in the model with that observed *in vivo* in human disease.

## 6. LIMITATIONS

The limitations of this analysis include assessment of expression by B2 and H6 4T1luc2 subclones of only the main E6 isoforms, i.e. *E6*I*, *E6*II* and the full-length E6 (*E6FL*), but not *E6*III* or *E6^E7*. Assessment of expression of additional E6 isoforms might have been more informative.

Expression of E6 and E7 was assessed only on the mRNA level, as commercially available anti-E6 and anti-E7 antibodies were found to be insufficiently sensitive to detect protein expression. Authors are currently working on generation of anti-E6 and anti-E7 antibodies detecting low (less than nanogram) amounts of E6 and E7, which would enable quantification of E6 and E7 protein expression in 4T1luc2 subclones in comparison with TC-1/luc2 cells. This would support the data on E6 and E7 expression based on the levels of respective mRNAs.

Tumor challenge experiments were done in groups of mice not exceeding five animals. The use of larger groups could have generated results of higher statistical significance. However, authors have chosen to minimize the number of animals to adhere to the 3R principles of animal research (replace, reduce, refine). The numbers used (4 to

5 animals per group) allowed us to obtain consistent results and fulfil the aims of the study. Besides, in all experiments, except for therapeutic immunization of E6 DNA-immunized mice with B2 and H6, these two subclones gave similar results, which allowed to pool the results obtained for B2 and H6 groups giving groups sized 8 to 10 animals.

Authors have not challenged mice with *E6/E7*-expressing 4T1luc2 cells with subsequent therapeutic DNA-immunization with E7 or E6/E7 combination. In the experiments we performed, both E7 and E6/E7 combination demonstrated poor results in the prophylactic setting and caused liver damage. DNA-immunization with E7 after challenge with *E6/E7*-expressing 4T1luc2 cells could have given additional proof to the observations on low immunogenicity of E7, and more data on the E7-related liver toxicity. This would have been informative, but was considered as not essential for the current study, and was not performed to reduce animal use and unnecessary animal suffering.

## 7. DESCRIPTION OF SUPPLEMENTARY MATERIALS

**Supplementary Figure S1** demonstrates consensus amino acid (aa) sequences of HPV16 oncoproteins E6 and E7 derived from HPV16 of Latvian isolates denoted by the GenBank accession numbers. Consensus was created by alignment of the translated nucleotide sequences using CLUSTAL MUSCLE algorithm of CLC Sequence Viewer 8.0 program (Qiagen, Aarhus, Denmark), sequence of HPV16 isolate NC_001526.4 served as a reference. Samples PQ215507 and PQ215505 contained a mixture of two HPV16 E6 variants with T6 and T6N. Samples PQ215490, PQ215495, PQ215504 contained a mixture of two HPV16 E6 variants, one with T6 and the other, with the aa substitution T6N. Labeled with the asterisk are entries containing given polymorphisms, with substitutions detected in > 30% of the reads.

**Supplementary Figure S2** illustrates the scheme of lentiviral vector pLJM1_pGK_E6E7 based on pLJM1-EGFP (https://www.addgene.org/19319/ ) with inserted DNA encoding E6 and E7 of the reference HPV16 isolate NC_001526.4. E6E7-coding sequences are placed under the control of the human EF1_alpha promoter cloned into pLJM1-EGFP in exchange for the original IE CMV promoter.

**Supplementary Figure S3** demonstrates the sensitivity of detection of HPV16 E6/E7 DNA by in-house and commercial PCR tests. Figure contains calibration curves built using Ct values in quantitative in-house PCR, quantitative commercial assay Allplex (Seegene, Seoul, South Korea) and semi-quantification commercial assay AnyPlex II HR HPV14 (Seegene). Assays were performed on a panel of samples containing known amount of lentiviral vector pLJM1_pGK_E6E7. Amount of plasmid DNA was recalculated into the copy number of E6/E7-encoding DNA. In-house PCR detected up-to 10 (Ct values below 32), AllPlex HPV HR Detection assay, up to a single copy of E6-encoding DNA, and semiquantitative AnyPlex II HPV HR Detection assay, 1 to 10 copies.

**Supplpementary Figure S4** shows results of validation of primers specific to E6, and reference genes *Gapdh* and *Actb* used to determine copy number of the genomic inserts of E6/E7 DNA by digital drop PCR (ddPCR), and determination of the copy numbers of E6 and E7 genes in relation to *Actb* and *Gapdh* in 4T1luc2 (contains none) and daughter clones 4T1luc2_EEB2 (B2) and 4T1luc2_EEH6 (H6).

**Supplementary Figure S5** illustrates the presence of infiltrating E6/E7-expressing 4Tluc2 cells, subclones B2 and H6, in the lungs, spleens and livers of the naïve BALB/c mice implanted with 4T1luc2_EEB2 (B2, n=4) and 4T1luc2_EEH6 (H6, n=4). Presence of tumor cells is characterized by the total photon flux [p/s] from mouse organs and by the threshold cycle of PCR (Ct) for DNA extracted from 10 sections of a FFPE block prepared from the respective mouse organ.

**Supplementary Figure S6** presents typical images (H&E staining, magnification x10) of the spleens and livers of BALB/c mice bearing tumors formed by subclones 4T1luc2_EEB2 (B2) and 4T1luc2_EEH6 (H6), with respective organs found positive or negative for the presence of tumor cells by both BLI and HPV16 E6-specific PCR. Images of the spleen visualize lymphoid follicles of various shapes, clearly expressed germinal centers and perifollicular areas. The stroma is edematous. Fibrosis or metastases are not visually detectable in either mice positive or negative for the presence of tumor cells by BLI and PCR. Images of the liver visualize rare accumulations of epithelioid cells with atypical nuclei (pathological mitoses) diffusely located between hepatocytes without signs of necrosis in mice negative for the presence of tumor cells by BLI and E6-specific PCR.

**Supplementary Figure S7** presents amino acid sequences of DNA-immunogens encoding HPV16 oncoproteins with amino acid substitutions introduced to abrogate their activity: pVaxE6-encoded E6 with C70G and I135T, and pVaxE7-encoded E7 with C24G and E26G. Sequences are aligned against amino acid sequences of E6 and E7 of the reference HPV16 strain NC_001526.4. Aligned to E6 and E7 are the sequences of peptides used for the analysis of specific immune response, with their abbreviated names.

**Supplementary Figure S8** demonstrates the results of the agarose gel electrophoresis of cDNA after RT-PCR of RNA isolated from 4T1luc2 transiently transfected with pVaxE6 and pVaxE7, with formation of specific bands - amplicons corresponding to E6 (79 bp), E7 (92 bp) and Hprt1 (155 bp), and the relative expression levels of E6 and E7 mRNA normalized to the expression of Hprt1, and calculated as fold change by ddCt method. Results represent three independent runs are presented as the mean ± SD.

**Supplementary Figure S9** demonstrates the absence of E6- or E7-specific triple IFN-g/IL-2/TNF-a cytokine secretion by CD4+ and CD8+ T cells of control mice immunized with pVax1 in response to stimulation with peptide pools representing E6 (E6/1, E6/2, E6/3) and individual peptides representing E7. Data represent group values as the mean ± SD. Statistical analysis was performed by Mann-Whitney test, *—$p < 0.05$.

**Supplementary Figure S10** illustrates an absence of the difference (Mann-Whitney test) in the weights of the tumors formed in mice prophylactically DNA-immunized E6, E7, E6/E7 and empty vector pVax1, by the E6/E7-expressing 4T1luc2 cells subclone B2 from the weights of the tumors formed by the E6/E7-expressing 4T1luc2 cells H6, by the experimental endpoint. All group values represent a mean ± SD. Absence of difference allows pooling data of B- and H6-challenged mice into one group.

**Supplementary Table S1** lists primers used for PCR to determine the number of copies of the provirus in the genome of the transduced lines, to clone E6/E7 region from pHPV16 plasmid into lentiviral vector, to perform PCR of 4T1luc2 cells transduced with lentiviral vector encoding E6/E7 to select subclones with E6/E7 DNA inserts, to perform ddPCR of subclones to determine the number of E6/E7 genomi9c inserts, to detect E6 or E7 DNA and DNA of the reference GAPDH gene in extracts of DNA from cell lines, murine tumors and tissues.

**Supplementary Table S2** presents data on the total flux from organs (lungs, spleen, liver) of E6, E7, E6/E7 and pVax1-immunized mice challenged with 4T1luc2_EEB2 (B2) and 4T1luc2_EEH6 (H6) subclones, showing no difference between the B2- and H6-challenged groups (all $p>0.05$ in Mann Whitney test), which allows to pool data obtained for the B2- and H6-challenged mice.

## 8. DECLARATIONS

### Ethics approval and consent to participate

A retrospective study of HR HPVs in the cervical tissues of women with high grade cervical lesions and cervical carcinomas diagnosed at the Paul Stradins University hospital was performed according to the ethical permit issued by the Ethical Committee of the Riga Stradins University N2-PĒK-4/415/2022 dated September 26, 2022. Informed consents to use biological materials in biomedical research were given by patients at the time of sampling.

Experiments in the laboratory mice were carried out in compliance with the bioethical principles adopted by the European Convention for the Protection of Vertebrate Animals Used for Experimental and Other Scientific Purposes (Strasbourg, 1986). Experimental procedures were approved by the Ethical Committee of the Latvian Food and Veterinary service (protocol nr 1.1-13/18/953), permit No 99 dated June 1, 2018, valid 01.06.2018-25.05.2023.

### Consent for publication

Manuscript does not contain any individual person's data in any form, such as individual details, images or videos, and does not require consent for publication.

### Availability of data and materials

All data underlying the results and conclusions of the manuscript are provided as figures and tables, and supplementary figures and tables. Raw data and materials used and/or analysed in the current study are available from the corresponding author upon reasonable request.

### Competing interests

Co-authors of this manuscript Dmitry Perminov and Ilze Lindenberga are employees of the E. Gulbja Laboratorija, Riga, Latvia, and performed analysis of the anonymized/blinded DNA samples according to the subcontract of Riga Stradins University, Riga, Latvia, financed by the project of the Latvian Science Fund 2021/1-0484. Maria Isaguliants is a member of the Editorial Board of "Infectious Agents and Cancer". Other authors declare no conflict of interest.

### Funding

- Project of the Latvian Science Fund LZP-2021/1-0484 "Human papillomavirus genome associated correlates of disease progression and treatment response for cervical neoplasms and cancer" to M.I. and J.N.;
- National Institutes of Health, USA, project NIHR01CA217715 "New therapeutic HPV vaccines for HIV infected men and women" to J.P. and S.C;
- International Academic Partnership Program: "EUROPARTNER: Strengthening and spreading international partnership activities of the Faculty of Biology and Environmental Protection for interdisciplinary research and innovation of the University of Lodz" of the Polish National Agency for Academic Exchange (NAWA), project number PPI/APM/2018/1/00007/U001, to M.I. and E.R. R.
- ERASMUS Internship is "Erasmus+ Programm über die Universität Hamburg", to H.N.

#### Authors' contributions

J.J. made substantial contributions to the conception and design of the work, data acquisition, analysis, and interpretation, participated in drafting and revision of the manuscript; D.A. made substantial contribution to the design of the work, data acquisition, analysis, and interpretation, drafted and revised the manuscript; A.D., E.R.R., L.S., D.P., I.L., H.N., S.G. participated in the acquisition, analysis and interpretation of the data; J.N. participated in the conception and design of the work, data acquisition, analysis, and interpretation; S.C. participated in the conception and design of the work, drafting and revision of the manuscript; D.S. participated in the conception and design of the work, data acquisition, and analysis; J.M.P. made substantial contribution to the conception and design of the work, interpretation of the data, participated in the revision of the manuscript; M.I. made substantial contributions to the conception and design of the work, data acquisition, analysis, and interpretation, drafted and reviewed of the manuscript. All authors have approved the submitted version.
